# Factors affecting the length of hospital stay for total knee arthroplasty in Japan: a retrospective study using the diagnosis procedure combination database

**DOI:** 10.1186/s40001-024-01714-w

**Published:** 2024-02-14

**Authors:** Kentaro Hara, Masato Kanda, Yoshio Kobayashi, Takashi Miyamoto, Takahiro Inoue

**Affiliations:** 1 Department of Operation Center, National Hospital Organization Nagasaki Medical Center, Nagasaki, 856-8562 Japan; 2 Department of Nursing, Nagasaki University Graduate School of Biomedical Sciences, Nagasaki, 852-8523 Japan; 3 Department of Cardiovascular Medicine, Chiba University Graduate School of Medicine, Chiba, 260-8677 Japan; 4 Department of Healthcare Management Research Center, Chiba University Hospital, 1-8-1 Inohana, Chuo-Ku, Chiba, Chiba 260-8677 Japan; 5 Department of Orthopedic Surgery, National Hospital Organization Nagasaki Medical Center, Nagasaki, 856-8562 Japan

**Keywords:** Length of hospital stay, Total knee arthroplasty, Diagnosis procedure combination, Orthopedic surgery

## Abstract

**Background:**

We aimed to evaluate the length of hospital stay following total knee arthroplasty to determine the impact of relevant factors using data from the Diagnosis Procedure Combination database.

**Methods:**

This was a retrospective observational study. The study cohort included 5,831 patients who had osteoarthritis of the knee and had undergone total knee replacement between February 2018 and October 2022 at 38 hospitals.

**Results:**

Multivariate analysis showed that the factors influencing the length of stay included: age (*p* < 0.001), height (*p* < 0.001), weight (*p* = 0.049), body mass index (*p* = 0.008), Barthel index (*p* < 0.001), method of anesthesia (*p* < 0.001), bone transplant (*p* = 0.010), timing of postoperative rehabilitation (*p* < 0.001), atrial fibrillation (*p* < 0.001), chronic pain (*p* < 0.001), and number of institutionally treated cases (*p* < 0.001) (*r* = 0.451, *p* < 0.001).

**Conclusions:**

Shorter or longer hospital stays were found to be associated with the patients’ background characteristics and facility-specific factors; these can lead to more accurate estimates of the length of hospital stay and appropriate allocation of resources.

## Background

Joint disease is the leading cause of concern for older adult patients needing support and care [1]. With the increase in the aging population worldwide, the incidence of osteoarthritis (OA) has been on the rise in recent years. Knee OA is characterized by cartilage destruction and synovial inflammation; it is a typical disease that impairs daily activities due to pain and a decreased motor function.

Total knee arthroplasty (TKA) is widely used for joint reconstruction not only in patients with end-stage knee OA, but also in patients with rheumatoid arthritis and advanced joint destruction. Annually, more than 30,000 patients in Japan undergo the procedure for pain relief and motor function improvement, and this number is only increasing every year [2]. In particular, a recent study predicted a further increase in the number of TKAs that would be performed by 2030 [3]. As a result, many healthcare system reforms are required to follow rehabilitation protocols for TKA to reduce the length of hospital stay. The length of hospital stay after TKA may depend on the patient’s background and social lifestyle, which require an accurate assessment [4]. In a study conducted in the United States of America, Leonie et al. successfully used the Risk Assessment and Prediction Tool (RAPT) to predict longer hospital stays after TKA and subsequently reduce the average hospital stay [5]. However, in some cases, RAPT predictions were found to be inaccurate [6, 7]. This may be because the RAPT score is evaluated only from the patient’s perspective, and includes the assessment of the following patient characteristics: age, gender, current walking status, and support status. We believe that assessment of more detailed patient information (such as the patient’s medical history) and institutional factors (such as the anesthetic procedure and facility information) may allow a more accurate prediction of the likelihood of an extended hospital stay. Moreover, many studies have shown that factors influencing the length of hospital stay after TKA include activities of daily living (ADL), patient factors, and pre-existing medical conditions [8, 9]; data on some of these factors are recorded in the Diagnosis Procedure Combination (DPC) database.

Therefore, this study aimed to determine the effect of relevant factors (with DPC data) on the length of hospital stay following TKA. We hypothesized that the post-TKA length of hospital stay depends on the patient’s admission and discharge information, background characteristics, comorbidities, and surgical information; these parameters are part of the DPC database. The findings of this study are expected to help in the estimation of the length of hospital stay of patients undergoing TKA based on the aforementioned factors.

## Methods

### Study design and ethical considerations

This multicenter, retrospective, observational study was conducted among healthcare institutions registered with the DPC and approved by the Ethics Committee of the Chiba University Hospital (approval no.: 3309). This study adhered to “strengthening the reporting of observational studies in epidemiology” guidelines [10]. A statistical plan was established before data were accessed, and analyses were performed after data collection [11]. The study was performed in accordance with the 1975 Declaration of Helsinki. Owing to the retrospective nature of the study, the requirement for informed consent was waived. Using the DPC database, data were collected regularly from hospitals included in the DPC system who volunteered to participate. The DPC database organizes administrative information obtained during acute-phase hospitalization and is used for reimbursement in the per-diem payment system. The database contains information on (but not limited to) the following: patient demographics (e.g., age, sex, height, and weight), the most resource-consuming disease, in-hospital deaths, other major diagnoses and comorbidities, consciousness level, ADL status, medications, treatment procedures, and other hospital-related information. Kanda and Inoue had full access to all the data in this study and take responsibility for data integrity and analysis. We published a research plan and presented participants with the opportunity to opt out online on our hospital’s homepage according to the instructions of the institutional review board.

### Study setting and population

This study was conducted from February 2018 to October 2022 in 38 hospitals with DPC data with which the Director’s Planning Office at the Chiba University Hospital (Chiba University School of Medicine) had a joint research agreement in Japan. Patients who were diagnosed with knee OA and had undergone TKA during hospitalization were included. The inclusion criteria were as follows: (1) patients undergoing orthopedic surgery, (2) patients of all ages, (3) patients of both sexes, (4) patients undergoing TKA, (5) patients undergoing general or spinal subarachnoid anesthesia, and (6) patients receiving any combination of epidural anesthesia. The exclusion criteria were as follows: (1) patients who underwent multi-TKA, (2) patients with missing data (e.g., smoking status, Barthel index), and (3) patients deemed inappropriate by the researchers.

### Outcome and data collection

The primary outcome was the length of hospital stay. We obtained the following data: (1) admission and discharge information (day of admission, day of discharge, and number of days in the hospital), (2) patient characteristics (sex, age, height, weight, body mass index [BMI], presence or absence of impaired consciousness, presence or absence of chemotherapy, use or absence of mechanical ventilation, and smoking status), (3) comorbidities on admission (diabetes mellitus, hypertension, atrial fibrillation, pneumonia, dyslipidemia, renal dysfunction, anemia, cerebrovascular disease, malignancy, venous thromboembolism, rheumatoid arthritis, osteoporosis, and chronic pain), (4) surgical information (surgical procedure, anesthetic method, and whether or not bone grafting was performed), (5) medical information (ADL status on admission and days to start rehabilitation), and (6) facility information (number of TKAs performed annually).

### Statistical analysis

The surveyed items, including patient characteristics, comorbidities on admission, surgical information, medical care information, and facility information, are tabulated, and their medians (quartile ranges) are shown. The data on the annual number of TKAs performed were divided into quartiles for statistical analysis. Multivariate analysis was performed for the primary and secondary endpoints, with the length of stay as the objective variable and the anesthesia method, patient background characteristics, comorbidities on admission, and Barthel index on admission as the explanatory variables. Differences with a significance level of 5% were considered statistically significant. JMP^®^ 15 (SAS Institute Inc., Cary, NC, USA) was used for all analyses.

## Results

### Patient background and facility information

**Fig. 1:**
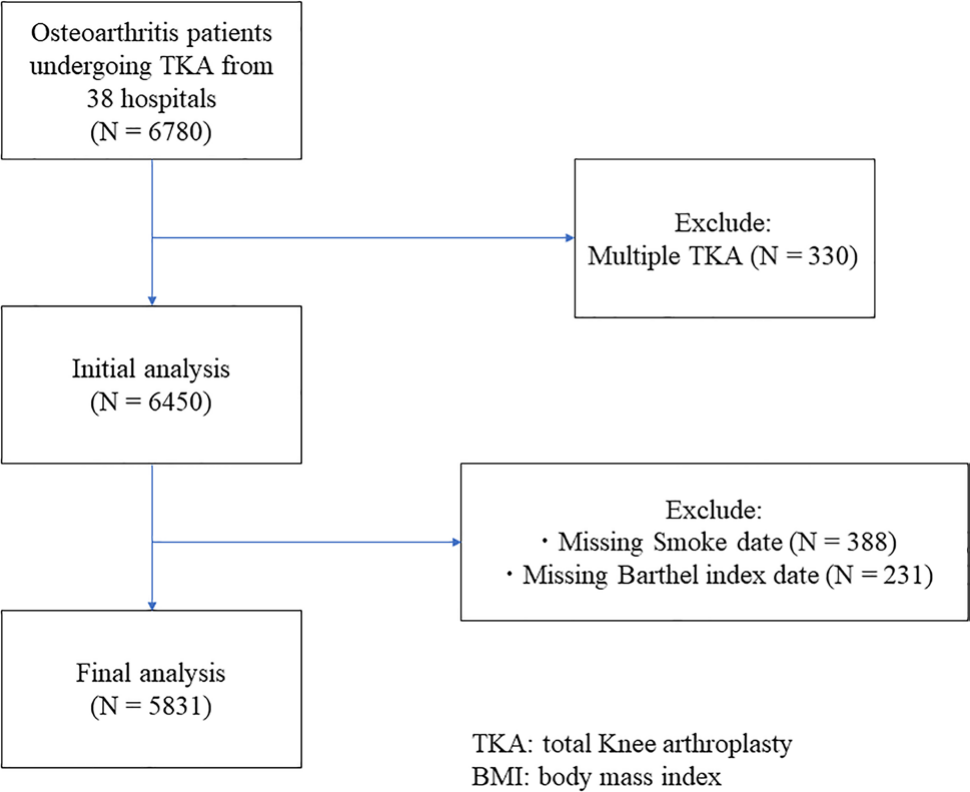
Trial STROBE diagram. Of the 6780 patients enrolled, 330 patients who underwent bilateral TKA and 619 with missing data were excluded; the remaining 5831 patients (1272 men and 4559 women) were included in the final analysis. STROBE: strengthening the reporting of observational studies in epidemiology, TKA, total knee arthroplasty, BMI: body mass index

Of the 6780 patients enrolled during the study period, 330 who underwent bilateral TKA and 619 with missing data (smoking status and the Barthel index) were excluded; thus, 5831 patients (1272 men and 4559 women) were included in the final analysis (Fig. 1).

Among these, 235 (4.0%) and 5596 (96.0%) patients were below and above 60 years of age, respectively. The Barthel indexes were < 20 in 20 patients (0.3%), 20–39 in 17 patients (0.3%), 40–59 in 83 patients (1.4%), 60–79 in 166 patients (2.8%), and ≥ 80 in 5545 patients (95.0%). General, epidural, and spinal subarachnoid anesthesia were induced in 1892 (60.0%), 34 (1.0%), and 385 (11.4%) patients respectively; furthermore, combined general and epidural anesthesia and combined spinal subarachnoid and epidural anesthesia were induced in 1000 (29.6%) and 70 (2.1%) patients, respectively (Table 1). The patients’ medical history is shown in Table 2.

**Table 1:** Patient characteristics and hospital information (N = 5831)

Sex	
Male	1272 (21.8%)
Female	4559 (78.2%)
Age (years)	
< 60	235 (4.0%)
60–64	329 (5.6%)
65–69	639 (11.0%)
70–74	1210 (20.8%)
75–79	1682 (28.8%)
80–84	1223 (21.0%)
85–89	562 (7.9%)
≥ 90	51 (0.8%)
Height (cm)	152 (147–158)
Weight (kg)	60.2 (53.4–68.0)
Body mass index (kg/m^2^)	
< 18.5	114 (2.0%)
18.5–24.9	1571 (27.0%)
25.0–29.9	3339 (57.3%)
30.0–39.9	771 (13.2%)
≥ 40.0	36 (0.6%)
Length of hospital stay (day)	21 (18–26)
Barthel index	
< 20	20 (0.3%)
20–39	17 (0.3%)
40–59	83 (1.4%)
60–79	166 (2.8%)
≥ 80	5545 (95.0%)
Smoking status	
Smoker	1163 (20.0%)
Non-smoker	4668 (80.0%)
Respirator	
Yes	155 (2.7%)
No	5676 (97.3%)
Anesthesia type	
General anesthesia	1892 (60.0%)
Epidural anesthesia	34 (1.0%)
Spinal anesthesia	385 (11.4%)
General and epidural anesthesia	1000 (29.6%)
Spinal and epidural anesthesia	70 (2.1%)
Bone transplant	
Yes	914 (15.7%)
No	4917 (84.3%)
Postoperative rehabilitation started (day)	3 (1–4)

Values are presented as medians (interquartile ranges) or numbers (percentages)

**Table 2:** Medical history (N = 5831)

Diabetes mellitus	
Yes	1267 (21.7%)
No	4564 (78.3%)
Hypertension	
Yes	1635 (28.0%)
No	4196 (72.0%)
Atrial fibrillation	
Yes	215 (3.7%)
No	5616 (96.3%)
Pneumonia	
Yes	5 (0.1%)
No	5826 (99.9%)
Hyperlipemia	
Yes	375 (11.1%)
No	3006 (88.9%)
Chronic kidney disease	
Yes	155 (2.7%)
No	5676 (97.3%)
Anemia	
Yes	607 (10.4%)
No	5224 (89.6%)
Cerebrovascular attack	
Yes	195 (3.3%)
No	5636 (96.7%)
Cancer	
Yes	167 (2.9%)
No	5664 (97.1%)
Pulmonary thromboembolism	
Yes	342 (5.9%)
No	5489 (94.1%)
Rheumatoid arthritis	
Yes	205 (3.5%)
No	5626 (96.5%)
Osteoporosis	
Yes	587 (10.1%)
No	5244 (89.9%)
Chronic pain	
Yes	515 (8.8%)
No	5316 (91.2%)

Values are presented as numbers (percentages)

The number of annual TKA cases (divided into quartile ranges) was 213 (3.7%) with fewer than 34 cases, 798 (13.7%) with 34–70 cases, 1445 (24.8%) with 71–104 cases, and 3375 (57.8%) with 104 or more cases (Table 3).

**Table 3:** Hospital information (N = 5831)

Annual hospital volume (case/year)	
< 34	213 (3.7%)
34–70	798 (13.7%)
71–104	1445 (24.8%)
> 104	3375 (57.8%)

Values are presented as numbers (percentages)

### Factors affecting the length of hospital stay

Multivariate analysis revealed that factors influencing the length of hospital stay were age (*p* < 0.001), height (*p* < 0.001), weight (*p* = 0.049), BMI (*p* = 0.008), Barthel index (*p* < 0.001), method of anesthesia (*p* < 0.001), bone transplant (*p* = 0.010), early postoperative rehabilitation (*p* < 0.001), atrial fibrillation (*p* < 0.001), chronic pain (*p* < 0.001), and number of institutionally treated cases (*p* < 0.001) (*r* = 0.451, *p* < 0. 001; Table 4).

**Table 4:** Multivariate analysis of factors associated with the length of hospitalization (N = 5831)

Factors	Partial regression coefficient	Standard error	*p*-value
Intercept	41.92	4.28	< 0.001*
Sex	0.16	0.22	0.476
Age (years)			< 0.001*
< 60	− 0.44	0.62	0.484
60–64	0.05	0.53	0.930
65–69	− 1.20	0.41	0.003*
70–74	− 0.54	0.33	0.100
75–79	− 0.02	0.30	0.927
80–84	1.31	0.33	0.001*
85–89	1.11	0.47	0.017*
> 90	− 0.24	1.23	0.839
Height	− 0.09	0.02	< 0.001*
Weight	0.03	0.02	0.049*
Body mass index (kg/m^2^)			0.008*
< 18.5	0.88	0.85	0.300
18.5–24.9	− 1.42	0.44	0.001*
25.0–29.9	− 0.63	0.41	0.121
30.0–40.0	− 1.02	0.48	0.033*
> 40.0	2.20	1.36	0.106
Barthel index			< 0.001*
< 19	− 6.58	1.84	< 0.001*
20–39	3.57	1.98	0.071
40–59	5.62	1.08	< 0.001*
60–79	− 0.51	0.91	0.572
≥ 80	− 2.09	0.71	0.003*
Smoking status	0.33	0.18	0.068
Respirator	− 0.23	0.40	0.552
Anesthesia type			< 0.001*
General anesthesia	− 0.61	0.41	0.139
Epidural anesthesia	− 2.61	1.19	0.028*
Spinal anesthesia	1.07	0.57	0.060
General and epidural anesthesia	0.71	0.43	0.104
Spinal and epidural anesthesia	1.44	0.91	0.116
Bone transplant	0.52	0.21	0.010*
Postoperative rehabilitation started	0.51	0.05	< 0.001*
Diabetes mellitus	− 0.12	0.16	0.428
Hypertension	0.28	0.15	0.064
Atrial fibrillation	1.21	0.34	< 0.001*
Pneumonia	− 1.06	2.21	0.630
Hyperlipemia	− 0.11	0.19	0.564
Chronic kidney disease	− 0.33	0.41	0.425
Anemia	0.01	0.22	0.991
Cerebrovascular attack	− 0.49	0.35	0.201
Cancer	0.13	0.39	0.728
Pulmonary thromboembolism	− 0.03	0.28	0.909
Rheumatoid arthritis	0.41	0.35	0.252
Osteoporosis	− 0.21	0.22	0.332
Chronic pain	0.52	0.23	0.026*
Annual hospital volume (case/year)			< 0.001*
< 34	6.41	0.52	< 0.001*
34–71	0.56	0.33	0.082
71–104	− 1.52	0.28	< 0.001*
> 104	− 5.46	0.25	< 0.001*

*Significant difference between the groups

## Discussion

Previous studies have shown RAPT to be useful for predicting the length of hospital stay after TKA. It comprises 12 items, including age, sex, walking distance, use of walking aids, and presence or absence of home care [5]. In line with previous findings, factors such as age and the Barthel index on admission were related to the duration of post-TKA hospitalization. Furthermore, novel factors, such as pain, differences in the anesthetic methods (surgical and anesthetic information), and presence or absence of comorbidities on admission, were also identified as being related to the duration of post-TKA hospitalization.

First, regarding the presence or absence of chronic pain, it is not uncommon for preoperative pain around the knee originating from the lumbar spine or hip to persist after TKA [12]. In addition, we also believe that chronic postsurgical pain (CPSP) after TKA should be considered. CPSP has been reported to occur in approximately 20% of the patients who undergo TKA [13]; pain after 2 months following TKA is often relieved by the subsequent course of therapy. However, there is a possibility that the pain may persist for 3–6 months or more after TKA [14, 15]. The Western Ontario and McMaster Universities Osteoarthritis Index (WOMAC), which allows patients to assess their physical health as a specific measure, has been widely adopted for patients undergoing prosthetic joint surgery. Previous studies have reported significantly higher scores on the pain, function, and stiffness scales of WOMAC during the preoperative assessment of patients who developed CPSP [16]. Since lower scores on the function and stiffness scales may cause a decline in the Barthel index, it was inferred that chronic pain and a low Barthel index on admission before surgery might lead to the onset of CPSP after TKA; this in turn would affect the postoperative hospital stay length. Further, studies have revealed shorter hospital stays among patients with a Barthel index of < 19; this may be due to their early transfers. However, in this study, only 0.1% of the patients had a Barthel index of < 19; because this sample size was small, further detailed analyses are required. In addition, height, weight, and BMI were significantly associated with the length of stay. Several studies have shown that maintaining an appropriate body weight lessened the burden on the knee joint, leading to a reduction in pain. Therefore, it is necessary to estimate the length of hospital stay, considering the increased risk of pain that continues after surgery in patients who do not have adequate weight control, have chronic pain before surgery, or have a low Barthel index on admission. Likewise, differences in the initiation of rehabilitation therapy after surgery affected the length of hospital stay. Therefore, it is important to understand the status of the Barthel index at the time of admission and start rehabilitation early.

Second, differences in the bone implants and anesthetic methods used had an impact on the length of stay. General and local anesthesia, including spinal subarachnoid anesthesia, are suitable for TKA; however, compared to general anesthesia, local anesthesia reportedly shortens the length of hospital stay by a greater extent [17–19]. This is consistent with the findings of the present study. General anesthesia has been reported to prolong the length of hospital stay in patients undergoing unilateral TKA [20, 21]; this is also consistent with our findings. However, contrastingly, some studies have shown that the method of anesthesia does not affect the length of hospital stay [22]. Nevertheless, studies have shown that patients who receive either epidural or spinal anesthesia while undergoing TKA always exhibit a difference in the postoperative pain; this is one of the reasons for the differences in the length of hospital stay [23, 24]. Although these previous studies are randomized controlled trials, we believe that they involve small sample sizes because they are single-center studies; further analyses of the effect of anesthesia methods on the length of hospital stay are needed to validate the results. In addition, bone transplantation was associated with the length of hospital stay in this study. This may be because patients who require bone transplants have poor knee conditions; such patients may take longer to rehabilitate. Therefore, patients who require bone transplantation should consider the possibility of experiencing longer hospital stays.

Third, atrial fibrillation (a comorbidity on admission) emerged as an associated factor for longer hospital stays in patients undergoing TKA. In a previous study, multivariate analysis of the length of hospital stay in patients with comorbid atrial fibrillation revealed the following as predictors of a longer hospital stay: acute coronary syndrome; acute decompensated heart failure; infection; heart failure with a reduced ejection fraction; heart failure with a preserved ejection function; elevated N-terminal-pro hormone brain natriuretic peptide levels; and elevated combinations of hypertension, abnormal liver/renal function, stroke history, bleeding history or predisposition, labile international normalized ratio, old age, and drug/alcohol usage [25]. In addition, frailty is common in patients with atrial fibrillation, which possibly impacts therapies and outcomes [26, 27]. Therefore, we inferred that patients undergoing TKA had suboptimal outcomes due to comorbidities, which may affect their length of hospital stay.

Fourth, the association with the annual number of TKA surgeries in an institution. Although the number of annual TKAs and length of hospital stay have not been previously reported. However, studies have revealed that the higher the annual number of surgeries, the higher the improvement in post-pancreatic cancer surgery outcomes and the lower the mid-term mortality rate after a coronary artery bypass surgery [28, 29]. This suggests that the higher the number of surgeries per year for TKA, the lower the complication rate and the shorter the hospital stay after surgery. We believe that this requires further analysis in the future; studies should compare the number of surgeries performed annually between facilities with high and low volumes of surgeries.

It is worth noting that accurately estimating the length of hospital stay for patients undergoing TKA can increase bed turnover in medical institutions and reduce patient wait times [30]. It can also reduce the cost of treatment, which can lead to a reduction in the burden of health insurance, cost of patient care, and savings in medical and health resources. In addition, an accurate estimation of the length of hospital stay may also reduce the potential risk of infection by reducing the probability of cross-infection among patients and shortening the time of direct contact between the patients and physicians [31]. In this study, we analyzed the patients’ backgrounds and facility information obtained from the DPC database and identified relevant factors that can influence the length of hospital stay. To our knowledge, this is the first study that used as many as 13 pieces of information on comorbidities on admission to estimate the length of hospital stays in patients undergoing TKA.

A notable strength of this study is its sample size. Several medical institutions in Japan have adopted DPC, which allowed for a large amount of data to be available for our analysis. In the future, we will further increase the sample size and perform similar analyses for different surgical procedures.

The challenges and limitations of this study were primarily related to the retrospective analyses of published databases. Attempts to statistically correct for biases have failed to rule out unmeasured and residual confounders that may have affected the length of hospital stay (e.g., usage and usage duration of different drugs and intraoperative blood loss). Furthermore, the hospital stay duration for our patients was relatively longer than that reported in Western countries; thus, caution must be exercised while applying our findings to different patient cohorts. In this study, the target institutions were limited to Japan; differences in the length of hospital stay between Japanese and international facilities may be attributed to the following: (1) differences in the clinical pathways set by each institution, (2) whether or not these pathways are used, (3) individual differences in the recovery process, and (4) differences in the medical culture. However, previous research suggests that the length of hospital stay in Japan is similar to that in some countries and will be further shortened due to changes in anesthesia and rehabilitation [32]. Therefore, we believe that the results obtained in this study may be generalized to other specific countries. Accordingly, it is necessary to validate our findings in prospective studies. Finally, the annual number of surgeries must be compared between high-volume and low-volume centers, and factors during hospitalization must be analyzed accordingly.

## Conclusions

Among the associated factors that can influence the length of hospital stay in patients undergoing TKAs, the age, height, weight, BMI, Barthel index, method of anesthesia, bone transplant, timing of postoperative rehabilitation, atrial fibrillation, chronic pain, and hospital volume of treated TKA cases are of primary importance and should be strongly considered during treatment.

## Data Availability

The datasets generated and/or analyzed during the current study are available from the corresponding author on reasonable request.

## Abbreviations

OA: Osteoarthritis
TKA: Total knee arthroplasty
RAPT: Risk assessment and prediction tool
ADL: Activities of daily living
DPC: Diagnosis procedure combination
BMI: Body mass index
CPSP: Chronic postsurgical pain
WOMAC: Western Ontario and McMaster Universities Osteoarthritis Index
